# Vasculitis in Systemic Autoinflammatory Diseases

**DOI:** 10.3389/fped.2018.00377

**Published:** 2018-12-03

**Authors:** Selcan Demir, Erdal Sag, Fatma Dedeoglu, Seza Ozen

**Affiliations:** ^1^Division of Pediatric Rheumatology, Department of Pediatrics, Hacettepe University Faculty of Medicine, Ankara, Turkey; ^2^Division of Immunology, Rheumatology Program, Boston Children's Hospital, Boston, MA, United States

**Keywords:** vasculitis, autoinflammatory diseases, inflammasomopathies, relopathies, interferonopathies, Behcet disease

## Abstract

Autoinflammatory diseases (AID) are diseases of the innate immune system, characterized by recurrent episodes of localized or systemic inflammation. Vasculitis may accompany AID. The causes of the association of vasculitis with monogenic AID are still debated. Among the monogenic AID, Familial Mediterranean Fever (FMF) is the most common. IgA-related vasculitis (IgAV) and Polyarteritis Nodosa (PAN) involving small and/or medium-sized vessels have an increased frequency among FMF patients. There are also case reports revealing vasculitic features in Cryopyrin-Associated Periodic Fever Syndrome (CAPS), Tumor Necrosis Factor Receptor-Associated Periodic Syndrome (TRAPS), Mevalonate Kinase Deficiency (MKD), also known as Hyper IgD syndrome (HIDS), Deficiency of IL-1 Receptor Antagonist (DIRA) and Pyogenic Arthritis, Pyoderma gangrenosum, and Acne (PAPA) patients. Central nervous system vasculitis and vasculopathy have been reported in DIRA and PAPA patients whereas small vessel involvement affecting skin has been reported in CAPS, TRAPS, and MKD patients. Alternatively, vasculitis can also be a leading feature especially in the recently defined monogenic AID (Otulipenia, Deficiency of Adenosine Deaminase 2-DADA2, Haploinsufficiency of A20) and interferonopathies (STING-associated vasculopathy with onset in infancy-SAVI). DADA2 often presents as a PAN-like disease. In otulipenia, patients have painful subcutaneous nodules caused by septal panniculitis with small and medium vessel vasculitis. Haploinsufficiency of A20 (also called Familial Behcet-like Autoinflammatory Syndrome) results in a phenotype very similar to the variable vessel vasculitis of Behcet's disease with recurrent oral-genital ulcers, in addition to, skin rash, uveitis, and polyarthritis. SAVI is an autoinflammatory vasculopathy with increased Interferon (IFN) signature, causing severe skin lesions resulting in ulceration, necrosis, and in some cases, amputation. Behcet's Disease (BD) is a multifactorial polygenic AID characterized by recurrent attacks of oral-genital ulcers, skin lesions, uveitis and a unique vasculitis affecting both arteries and veins of all sizes. Many clinical features overlap with other autoinflammatory diseases and overexpression of proinflammatory cytokines is an important feature of the disease.

## Introduction

The first term used for a self-reacting immune-system was “horror autotoxicus” suggested by Paul Ehrlich (1). With the definition of autoinflammatory diseases, Dr. Kastner's group suggested the term “Horror Autoinflamaticus” for the autoinflammatory disease state, where the self-reaction is in the form of inflammation (2). At that time, the autoinflammatory diseases (AID) were described as unprovoked episodes of systemic inflammation due to abnormal activation of the innate immune system without high-titer autoantibodies and antigen specific T and B cells (2). These unique episodes generally occur periodically with fever and other systemic manifestations such as rash, arthritis, serositis, lymphadenopathy, central nervous system, and other organ involvements. AID can either be monogenic in nature due to a mutated single causative gene (FMF, CAPS, TRAPS, etc.) or a multifactorial polygenic disease such as Behcet's disease.

**Figure 1:**
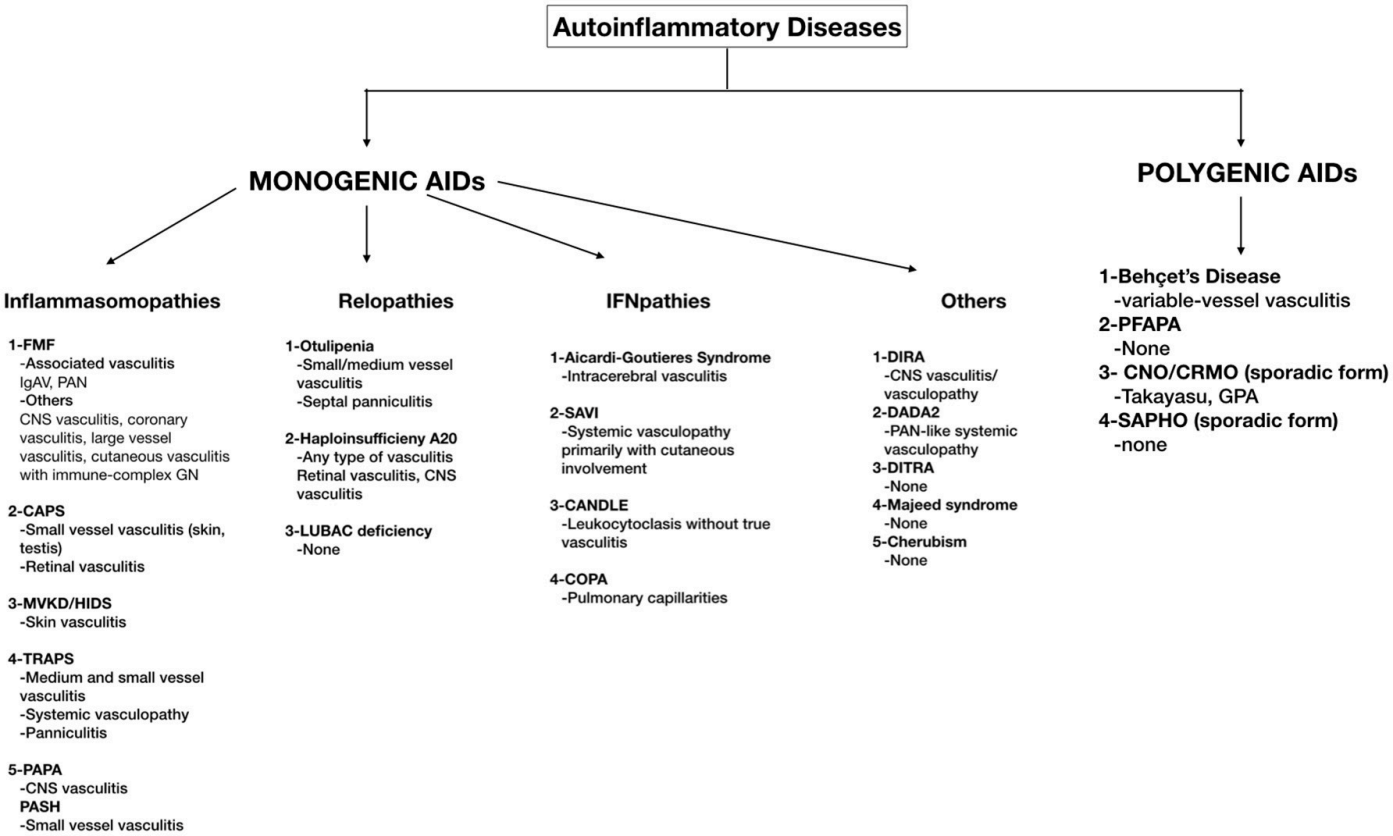
A schema of monogenic and polygenic autoinflammatory diseases with vasculitic features. FMF, Familial Mediterranean Fever; IgAV, Immunoglobulin A vasculitis; PAN, Polyarteritis Nodosa; CNS, Central Nervous System; GN, Glomerulonephritis; CAPS, Cryopyrin Associated Periodic Fever Syndrome; MKD/HIDS, Mevalonate kinase deficiency/Hyperimmunoglobulin D syndrome; TRAPS, Tumor Necrosis Factor Receptor–Associated Periodic Syndrome; PAPA, Pyogenic Arthritis, Pyoderma gangrenosum; PASH, Acne Pyoderma gangrenosum; Acne, Hidradenitis Supurativa; LUBAC, Linear ubiquitin chain assembly complex; SAVI, STING-associated vasculopathy with onset in infancy; CANDLE, Chronic atypical neutrophilic dermatosis with lipodystrophy and elevated temperature syndrome; DIRA, Deficiency of Interleukin-1 Receptor Antagonist; DADA2, Deficiency of Adenosine Deaminase-2 (DADA2); DITRA, deficiency of the interleukin-36 receptor antagonist; PFAPA, periodic fevers with aphthous stomatitis, pharyngitis, and adenitis; CNO/CRMO, Chronic nonbacterial osteomyelitis/Chronic recurrent multifocal osteomyelitis; SAPHO, Synovitis, Acne, Pustulosis, Hyperostosis, and Osteitis.

Vasculitis is inflammation of the blood vessel wall, generally categorized by the predominant type of vessels involved, not only in size (small vessel, medium vessel, large vessel, and variable vessel vasculitis), but also in structural and functional attributes (single organ vasculitis, vasculitis associated with systemic disease and vasculitis associated with probable etiology) (3). Vasculitis/vasculopathy in various forms is associated with many types of immune-mediated conditions such as autoimmune diseases and immunodeficiencies, as well as, a variety of infectious diseases. Autoinflammatory diseases are no exception. Generally, the heightened and spontaneous inflammatory response in autoinflammation is due to the insufficient or absent break mechanism of immune response, in which vessels seem to be commonly affected resulting in variety of vasculitis. Vasculitidies are among the differential diagnoses for autoinflammatory diseases as they share common features such as fever and skin involvement. Furthermore, vasculitis can also be either one of the features of or highly associated with an autoinflammatory disease (4, 5). In this review, we will discuss the vasculitic features of both monogenic AID and BD, as a multifactorial/polygenic AID (Figure 1).

## Monogenic AID

### Inflammasomopathies

Whether vasculitis in inflammasomopathies is a true component of the disease or it is an association is still controversial. Chen et al. demonstrated in a Kawasaki mouse model that NLRP3 inflammasome activation was associated with development of coronary arteritis (6). In another mouse model, it has been observed that infusion of visfatin, a major injurious adipokine during obesity, increased NLRP3 inflammasome formation and IL-1 production leading to enhanced endothelial inflammatory response and endothelial dysfunction (7). However, the molecular mechanisms of the vasculitic features of inflammasomopathies are far from clear.

On the other hand, some vasculitides are indeed more common among these patients. It has been suggested that this is due to the enhanced innate immune response in these patients (8). However, occasional vasculitic features have been shown in Tumor Necrosis Factor Receptor-Associated Periodic Syndrome (TRAPS) (see below).

#### Familial mediterranean fever (FMF)

**Figure 2:**
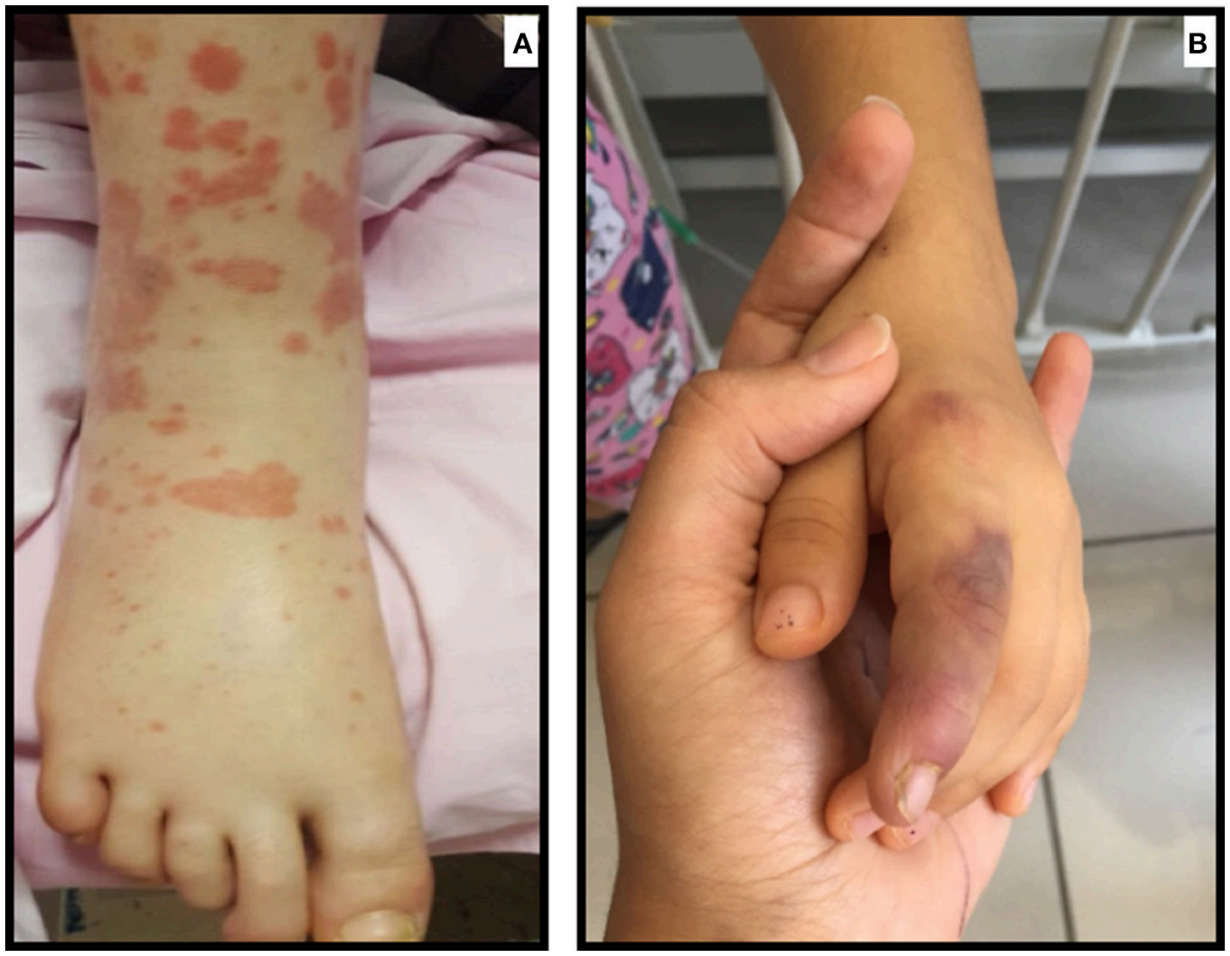
**(A)** Atypical purpuric rash in a patient with FMF who developed IgAV/HSP **(B)** Ischemic lesion due to medium-size vessel involvement in a child with FMF who developed PAN.

FMF is the most common AID in childhood. It is characterized by recurrent febrile episodes with serositis, synovitis, and erysipelas-like erythema (ELE), which usually subside within 24–72 h. The disease is associated with the *MEFV* mutations encoding a protein called “Pyrin” (9). ELE is the characteristic skin rash in FMF patients; however, the histopathologic features of these lesions are not consistent with vasculitis (10). The most common vasculitis associated with FMF, with a frequency of 1–7%, is Immunoglobulin A-associated vasculitis (IgAV or otherwise known as Henoch Schönlein Purpura) (11–14) (Figure 2A). *MEFV* mutation has also been shown to be more common among IgAV patients (15, 16). IgAV in FMF patients has a different course; it tends to reoccur several times, is seen in younger children, and the rash can develop in unusual locations such as the face and trunk (17). In studies where a biopsy was available, leukocytoclastic vasculitis without IgA deposition was shown (17). Ben-Chetrit et al. suggested that vasculitis similar to IgAV seen in FMF is a distinct feature of FMF itself (17). However, it has also been suggested that certain inflammatory diseases are more frequent in FMF because of the exaggerated immune response (8).

Another common vasculitis seen in FMF is PAN (polyarteritis nodosa), which can be found in 1% of FMF patients (11, 14). PAN in FMF tends to have a younger-onset, more frequent perirenal hematomas, more severe myalgia, and an overall better prognosis (18) (Figure 2B). Elevated Antistreptolysin-O, but not hepatitis B antigen titer, was seen in PAN-FMF patients (18). There is still no consensus on whether PAN seen in FMF is coincidental or directly associated (18).

Besides these common vasculitic manifestations, there are some case reports of FMF patients with central nervous system involvement (19, 20), coronary vasculitis (21), large vessel vasculitis (similar to Takayasu arteritis) (22), and cutaneous vasculitis with immune-complex glomerulonephritis (23).

Treatment with high-dose corticosteroids along with immunosuppressants, as deemed necessary, have been used. Although FMF is a disease with multiple exacerbations of fever, skin rash, and other symptoms, interestingly, most reports and series of vasculitis described single episodes often responding well to immunosuppressive therapy (24).

#### Cryopyrin associated periodic fever syndrome (CAPS)

CAPS is a rare AID caused by a mutation in *CIAS1* gene and is characterized by periodic fever, urticarial rash, arthritis, conjunctivitis, and neurological involvement, including hearing loss and aseptic meningitis. It was historically considered as three distinct clinical diseases: Familial Cold Autoinflammatory syndrome (FCAS), Muckle-Wells Syndrome (MWS), and Chronic Infantile Neurological Cutaneous and Articular Syndrome (CINCA)/Neonatal-onset Multisystem Inflammatory Disorder (NOMID), but is now recognized as a severity spectrum rather than separate entities (25). The typical skin lesion is neutrophilic urticarial dermatosis with perivascular and peri-eccrine infiltrates without evidence of vasculitis (26). There are only case reports revealing small vessel vasculitis of skin and testis (27) and also retinal vasculitis (28, 29) responsive to corticosteroid and azathioprine treatments. Therefore, vasculitis is not regarded as an integral part of CAPS but can be seen as a coexisting disease.

#### Mevalonate kinase deficiency/hyperimmunoglobulin D syndrome (MKD/HIDS)

HIDS is characterized by recurrent fever, rash, lymphadenopathy, gastrointestinal manifestations and, in severe forms, neurological abnormalities, and ocular and auditory findings (30). The most common types of rash are erythematous macules, papules, urticarial lesions, and erythematous nodules (31). The original article, van Der Meer et al. reported that the skin biopsy of a patient during attack had lymphocytic vasculitis with mononuclear infiltrates around vessels high and deep in the dermis, swelling of the endothelium, extravasation of erythrocytes, and nuclear detritus (32). In another cohort of 44 HIDS patients, 10 of them underwent skin biopsy which showed mild features of vasculitis. Non-specific findings were noted in five biopsy specimens; sweet-like neutrophilic dermatosis in two, cellulitis-like findings in one, and deep vasculitis characteristics in another (31). There are also other case reports showing vasculitis in skin biopsies, with IgD and C3 depositions in one (33, 34). Therefore, skin vasculitis can be a component of HIDS.

#### Tumor necrosis factor receptor–associated periodic syndrome (TRAPS)

Tumor Necrosis Factor Receptor Associated Periodic Syndrome (TRAPS) is an autosomal dominant disease, caused by mutations in the *TNFRSF1A* gene. It is characterized by recurrent febrile episodes (lasting >1 week), serositis, periorbital edema, and myalgia with overlying migratory rash (25).

In a group of 10 TRAPS patients with skin biopsy, microscopic examination showed a superficial and deep perivascular and interstitial infiltrate of lymphocytes and monocytes without a clear evidence of multinucleated macrophages, granulomatous, or leukocytoclastic vasculitis (35). Lamprecht et al. reported 2 adult patients, one who had small vessel vasculitis and panniculitis with ANCA positivity against human leukocyte elastase, and another who had fasciitis and panniculitis (36). Another adult study from Spain reported leukocytoclastic vasculitis in 1 out of 6 TRAPS patients (37). Cammelli et al. reported the autopsy findings of a 26-year-old TRAPS patient that showed pronounced intimal thickening and medial hypertrophy of medium and small vessel walls without inflammatory cell involvement suggesting a systemic vasculopathy (38). Therefore, vasculitic features may accompany TRAPS.

#### Pyogenic arthritis, pyoderma gangrenosum, and acne (PAPA)/pyoderma gangrenosum, acne, hidradenitis supurativa (PASH)

PAPA is an AID caused by *PSTPIP1* gene mutation and is characterized by arthritis, severe cystic acne, and pyoderma gangrenosum (39). Hidradenitis supurativa is the characteristic feature of PASH. The skin biopsy of a PASH patient revealed a perivascular, interstitial infiltrate composed of neutrophils, demonstrating prominent leukocytoclasis, extravasation of red blood cells, and focal fibrin deposition within small vessel walls (40). Another case report of a patient with PAPA syndrome reported an unusual cerebral arterial vasculopathy/vasculitis that resulted in a subarachnoid hemorrhage from a ruptured dissecting posterior cerebral artery aneurysm, which was treated with an endovascular coil. However, it was not clear if PAPA syndrome itself caused the cerebral vasculitis, or if it was a co-existing condition (41).

### Relopathies/nuclear factor kappa-B (NF-κB) related AID

The nuclear factor κB (NF-κB) pathway is tightly regulated through multiple posttranslational mechanisms, including ubiquitination. Linear ubiquitin chain assembly complex (LUBAC) mediated ubiquitination induces the NF-κB pathway; however, deubiquitinases such as A20, OTULIN, CYLD, and Cezanne, function as negative regulators of NF-κB signaling. LUBAC is a trimer composed of HOIL-1, HOIP, and SHARPIN (42, 43). Decreased expression of LUBAC complex subunits, in patients who carry mutations in HOIP or HOIL-1, results in inhibition of the NF-κB pathway in fibroblasts and B-cells, thereby causing immunodeficiency. The monocytes of these patients produce proinflammatory cytokines (43). A secondary vasculitis may be a part of these diseases; it is speculated that the NF-κB pathway is operative on inflammation in the endothelial cell. It was demonstrated in murine models that, the deficiency of the catalytic HOIP subunit of LUBAC leads to embryonic lethality at midgestation due to endothelial cell death and vascularization defect mediated by TNFR1 (44).

#### Otulipenia

Otulipenia is the result of the loss-of-function mutation of *OTULIN*, which encodes a deubiquitinase, causing an increase in canonical NF-κB pathway activation, accumulation of poly-ubiquitinated *OTULIN* target proteins, e.g., NEMO, RIPK1, ASC, TNFR1, and elevated levels of secreted proinflammatory cytokines, such as IL-6 and TNF (45). This paper reported 4 patients with this peculiar disease having neonatal-onset recurrent fever, neutrophilic dermatitis/panniculitis, lipodystrophy, and failure to thrive, but without obvious primary immunodeficiency (45). Skin biopsy of one of the patients revealed septal panniculitis and vasculitis of small and medium sized vessels (45).

#### Haploinsufficency of A20 (HA20)

Recently, Zhou et al. described a new AID caused by heterozygous loss-of-function mutations in *TNFAIP3*, leading to haploinsufficiency of A20 (HA20) and resulting in a very similar phenotype with variable-vessel vasculitis-Behcet's disease (46). Patients presented with early-onset, recurrent oral and genital ulcers, gastrointestinal manifestations, fever, polyarthralgia/arthritis, skin involvement, and cardiac and ocular manifestations. A review of 16 HA20 patients revealed retinal vasculitis in one patient and CNS vasculitis in another (diagnosed with brain imaging). In the third patient, CNS vasculitis (detected by frontal lobe biopsy) plus pulmonary artery emboli were found (47).

### Interferonopathies

Type I interferonopathies comprise a group of heterogeneous autoinflammatory diseases associated with constitutive activation of type I interferon signaling. The intrinsic dysregulation of the interferon pathway affects the vasculature and results in vasculopathic features in these patients.

#### Aicardi–goutières syndrome

Aicardi–Goutières syndrome is an inflammatory disease that occurs due to mutations in any of the *TREX1, RNASEH2A, RNASEH2B, RNASEH2C, SAMHD1, ADAR*, or *IFIH1* genes. It causes a phenotype similar to intrauterine viral infections such as congenital encephalopathy, basal ganglia calcifications, hepatomegaly, glaucoma, hypothyroidism, cardiomyopathy, and a demyelinating peripheral neuropathy. The interferon and IFN-related proteins are increased in both the peripheral blood and cerebrospinal fluid (48). From the largest cohort of 374 patients, intracerebral vasculitis was seen frequently enough to be confirmed as being associated with the Aicardi-Goutieres syndrome phenotype, especially in the patients with *SAMHD1* mutations (48).

#### STING-associated vasculopathy with onset in infancy (SAVI)

*SAVI* is an autoinflammatory disease caused by gain-of-function mutations in *TMEM173* (49). The disease is characterized by recurrent fevers, interstitial lung disease, increased inflammatory parameters, and failure to thrive; however, the hallmark of the disease, as its name applies, is the vasculopathy. Cutaneous manifestations were the most striking features of vascular changes. The nail fold capillary tortuosity and capillary loss in the early phase of the disease may lead to scarring of the acral areas, such as ear cartilage, and perforation of the nasal septum, dystrophic nail changes, resorption of distal phalanges of fingers and toes, and gangrenous digits, eventually necessitating surgical amputation (49).

#### Chronic atypical neutrophilic dermatosis with lipodystrophy and elevated temperature (CANDLE) syndrome

CANDLE syndrome is another interferonopathy caused by mutations in *PSMB8* resulting in dysfunctional proteasomes, which is also termed as one of the proteasome-associated autoinflammatory syndromes (PRAAS). Nakajo-Nishimura syndrome-NKJO (nodular erythema, elongated and thickened fingers, emaciation, large eyes, nose, lips, and ears, and loss of adipose tissue in the upper part of the body), and JMP syndrome (joint contractures, muscular atrophy, microcytic anemia, and panniculitis-induced lipodystrophy) are the other conditions under the PRAAS umbrella. The clinical features of CANDLE syndrome manifest as early-onset recurrent fevers, annular violaceous plaques, persistent violaceous eyelid swelling, failure to thrive, lipodystrophy, hepatomegaly, joint involvement, interstitial lung disease with a couple of specific laboratory abnormalities including chronic anemia, elevated acute-phase reactants and raised liver enzymes (50, 51). Skin biopsies of CANDLE patients revealed perivascular and interstitial dermal infiltrates extending into the subcutaneous tissue. Leukocytoclasis was often present, but true vasculitis with fibrinoid necrosis of the vessel walls has not been reported (52).

#### COPA syndrome

COPA syndrome is a recently defined disorder of immune system which has been associated with type 1 interferon activation (53) This disease is an autosomal dominant inherited disease and caused by mutations in COPA gene, which encodes alpha subunit of coatomer protein complex I (COPα). COPα regulates the transition of proteins between the Golgi and the endoplasmic reticulum. The mutant COPA gene causes an increased endoplasmic reticulum stress due to impaired intracellular transport (54). Main features of Copa Syndrome include erosive polyarthritis, interstitial lung disease with or without pulmonary hemorrhage and kidney disease with autoantibodies (ANA, ANCA, RF, and others). Histopathologically, increased red blood cells and hemosiderin-laden macrophages were present in the alveolar spaces. Signs of pulmonary capillaritis were evident in most subjects with necrosis of the capillary walls. Neutrophils were often identified along the capillaries, consistent with immune-mediated hemorrhage (55).

In interferonopathies, JAK1/2 inhibition with baricitinib treatment showed promising results; it improved the clinical manifestations and inflammatory and IFN biomarkers in patients with CANDLE, SAVI, and other interferonopathies (56).

### Other AID

#### Deficiency of interleukin-1 receptor antagonist (DIRA)

DIRA is a very rare autoinflammatory syndrome primarily affecting the skin and bone and is caused by recessive mutations in IL1RN, the gene encoding the interleukin-1–receptor antagonist (57). Patients present in the neonatal period with severe neutrophilic “pustular” skin eruption, nail dystrophy, sterile osteomyelitis, and periostitis. In the original article, Aksentijevich et al. reported vasculitis in the connective and fat tissue adjacent to bone in the biopsy, and central nervous system vasculitis/vasculopathy on MRI in 1 out of 9 patients (57). The vascular features may be explained by an animal model: In the IL-1Ra (–/–) mice model, IL-1Ra deficiency in T cells activates them excessively, resulting in the development of aortitis in a TNF-alpha-dependent manner (58).

Vasculitis does not seem to be a common feature in other AIDs affecting bones such as CNO (chronic non-bacterial osteomyelitis), Majeed syndrome, SAPHO (synovitis, acne, pustulosis, hyperostosis, and osteitis), DITRA (Interleukin-36 receptor antagonist deficiency) and Cherubism, except that case reports of CNO patients with associated granulomatosis with polyangiitis (GPA) and Takayasu arteritis have been reported (59, 60).

#### Deficiency of adenosine deaminase-2 (DADA2)

DADA2 is another newly defined disease similar to polyarteritis nodosa (PAN) with recurrent stroke, livedoid skin rash, and immunodeficiency features. The most striking feature of the disease is vasculopathy. The reader is referred to the review on DADA2 in this issue and thus, we will not discuss it further.

## Polygenic AID

### Behcet's disease

Behçet Disease (BD) is a multigenic autoinflammatory disorder of unknown etiology. It is a multisystemic disease and characterized by recurrent mucocutaneous, ocular, musculoskeletal, vascular, gastrointestinal, and central nervous system manifestations. It was first described in 1937 by a Turkish dermatologist, Hulusi Behçet (61). BD can affect arteries and veins of any size and thus, it has been classified as “variable vessel vasculitis” in International Chapell Hill Consensus Conference (CHCC 2012) (3).

Behçet Disease is more common along the historic “Silk Road” from Eastern Asia to the Mediterranean (62). Turkey has the highest prevalence rate (up to 420 per 100,000) (63). In case studies, BD is equally prevalent among male and female populations; however, the disease course is more severe in males (64). The diagnosis is based mainly on clinical findings and there is no specific diagnostic test. Due to the heterogenic clinical presentation of (BD), many different diagnostic criteria have been proposed to date. The International Study Group (ISG) diagnostic/classification criterion for BD is widely used (65). However, the ISG criteria do not involve vascular manifestations and it fails to classify most of pediatric BD patients. Recently, an international expert consensus group (the pediatric BD group) developed a new classification criterion for pediatric Behçet's disease (66). The performance of the new pediatric criteria was found to be 73.5% sensitive and 98.9% specific by Turkish and Israel groups (67). Currently, the Outcome Measures in Rheumatology (OMERACT) BD study group is working to develop a core set of outcome measures for BD, especially for vascular BD (68).

BD has similar clinical features with monogenic autoinflammatory disorders. Because of the absence of autoimmune T and B cells in pathogenesis, the episodic nature of the disease course, and the abnormally increased inflammatory response, BD has been considered as a polygenic autoinflammatory disease (69). It is suggested that environmental factors such as microbial agents may trigger the inflammatory episodes by inducing an aberrant immune response in genetically susceptible individuals (70). Recently Ambrose et al. showed over-expression of CXCL10 protein by IFN-γ stimulation in BD monocytes. They suggested that dysfunctional posttranscriptional regulation of CXCL10 mRNA, resulting in increased CXCL10 protein levels may contribute the exaggerated inflammation in BD (71). Despite the identification of newer genetic associations, HLA-B51 is still the most important genetic link affecting the disease risk and typical phenotype (72). BD has a complex genetic background in pathogenesis. Large genome-wide association studies (GWAS) have confirmed the association of BD with HLA–B51/B5 and have also shown an association with *interleukin (IL)-10*, and *IL23R/IL12RB2, STAT4, endoplasmic reticulum aminopeptidase1 (ERAP1)* genes (73–78). IL-10 is an anti-inflammatory cytokine, IL-23 has a role in inflammatory Th17 pathway and STAT4 is a transcription factor in signaling pathways related to IL-12, Type 1 IFN, and IL-23. ERAP1 is an aminopeptidase expressed in endoplasmic reticulum and is responsible for processing and loading peptides onto MHC class I molecules. Two single nucleotide polymorphisms (SNPs) in ERAP1 were found in association with BD and confirmed in different ethnic groups (76). The presence of SNPs in ERAP1 cause an unfolded protein response and endoplasmic reticulum stress triggering inflammation through the IL-23/IL17 pathway in BD (79, 80). Furthermore, GWAS described an epistatic interaction in the ERAP1 and MHC class I region. It has been shown that homozygosity for the ERAP1 variants is associated with an increased risk in HLA-B51 positive patients (76). Non-HLA genetic associations, such as ERAP1, IL23R, and IL10 variations, suggest that BD has some common susceptibility genes and inflammatory pathways with spondyloarthritis (70). However, this pathway fails to explain the vasculitis of BD.

In fact, vascular involvement is one of the major causes of morbidity and mortality in Behçet's disease and is more common and severe in males, particularly from the Middle East and Eastern Mediterranean (80, 81). Vascular involvement occurs up to 40% of patients with BD, 75% of them are presenting as venous involvement while 25% are arterial (64). Vascular involvement in BD has a relapsing course. In a recent study from Turkey, the recurrence rate of vascular events in BD was reported as 34.5%. A new vascular event risk was 23% at 2 years and 38.4% at 5 years (82). In the vascular cluster of BD, several types of vascular involvement may be seen together (82). There is an association between cerebral sinus venous thrombosis (CSVT) and pulmonary artery involvement (PAI) (82), intracardiac thrombosis and PAI (83), Budd-Chiari syndrome (BCS), and inferior vena cava syndrome (84). Lower extremity vein thrombosis (LEVT) often accompanies these combinations and often occurs prior to the other vascular involvements.

The vasculitis of BD has unique features. The involvement of the venous system is frequent and is almost always in the form of thrombosis. However, the thrombosis of BD does not result in emboli. It is also noteworthy that the arterial inflammation has not been associated with atherosclerosis.

The mechanism underlying the thrombosis in BD remains poorly understood and there are a number of studies in this field. Recently Khan et al. have compared the blood plasma of 72 healthy controls with 88 BD patients with or without thrombosis. They found that BD patients had significantly higher numbers of microparticles (MP) and MP expressing tissue factor (TF) and tissue factor pathway inhibitor (TFPI) than healthy controls. Furthermore, BD patients with thrombosis had increased levels of TF positive MP and decreased levels of TFPI positive MPs. They suggested that the intrinsic imbalance in the endothelium of the vessels results in the risk of thrombosis in BD (85). As previously mentioned, venous disease is more common than arterial involvement (86, 87). LEVT is the most frequent manifestation (86). BD associated LEVT has discriminative features when compared to LEVT due to other reasons. It has been shown that they had significantly more bilateral involvement, less complete recanalization, and more frequent collateral formation (88). Due to the extension of the LEVT, the most involved part of VCI is the infrahepatic part. Hepatic and suprahepatic VCI thrombosis cause Budd-Chiari syndrome (84). CSVT is common among children and it occurs generally shortly after the onset of the disease (89). Superior sagittal and transverse sinuses are frequently affected. CSVT is associated with other vascular involvements in the body, such as LEVT (90). LEVT and CSVT may be the early signs of other thrombi that will develop in the body. Superior and inferior vena cava occlusion, Budd-Chiari syndrome, cerebral sinus thrombosis, and other venous obstructive lesions are other common manifestations of involvement on the venous side (82, 91). Recurrent thrombosis of the lower extremities may cause post-thrombophlebitic syndrome (88).

Pulmonary artery involvement (PAI) is the most common form of arterial involvement in BD (92). Aortic, iliac, femoral, popliteal, and carotid arteries are the other commonly affected arteries. Although the most common arterial pathology is aneurysm, occlusion and stenosis with thrombi of the aorta, femoral, or pulmonary vessels can also occur (93).

PAI is strongly associated with LEVT, CSVT, and intracardiac thrombosis and it develops generally in the early stages of the disease (92). In a recent study, it has been shown that patients with extra-pulmonary arterial involvement were significantly older than the patients with PAI (82).

Pulmonary artery aneurysm (PAA) is an important cause of morbidity and mortality in BD and hemoptysis is the most commonly presenting symptom. The other significant type of PAI is pulmonary arterial thrombus (PAT). In a study of 47 BD patients with PAI, 72% presented with PAA with or without PAT and the remaining 28% with isolated PAT (83). The most common presentation of cardiac involvement is intracardiac thrombosis and is associated strongly with PAI (83, 94). Coronary artery vasculitis is not common but important because it may lead to acute myocardial infarction. Atherosclerosis does not increase appreciably in (BD) (95). However, a recent meta-analysis showed that flow-mediated inflammation was impaired and intima media thickness increased in BD, thereby causing an increase of subclinical atherosclerosis in BD (96).

EULAR recommendations of the BD management were updated in 2018 (97). According to the new recommendations, glucocorticoids and immunosuppressives, such as azathioprine, cyclophosphamide, or cyclosporine-A, are recommended for the management of acute deep vein thrombosis. For pulmonary artery aneurysms, high-dose glucocorticoids and cyclophosphamide are recommended as treatment options. In refractory patients, monoclonal anti-TNF antibodies could be considered and if the risk of bleeding is low, anticoagulants may be added, except for the patients with pulmonary artery aneurysms. For patients who have a high risk of bleeding, embolization should be the preferred treatment rather than open surgery. For both aortic and peripheral artery aneurysms, medical treatment with cyclophosphamide and corticosteroids is necessary before surgery; however, if the patient is symptomatic, surgery, or stenting should not be delayed. For the first episode of cerebral venous thrombosis, high-dose glucocorticoids are recommended to obtain rapid remission. Additionally, anticoagulants may be beneficial in patients who have an additional prothrombotic tendency.

## Conclusion

Vasculitis can be a coexisting disease seen with AID. Vasculitis may also be one of the features of an AID and can even be the most striking feature of certain types of AID, such as Behcet's Disease, DADA2, SAVI, AGS, and HA20. The underlying pathology is still not clear. Increased IL-1, IFN, or immune-complexes may be the cause of endothelial damage. The more we understand the pathophysiology of vasculitis and AIDs, the better we can define the connections between these two conditions.

## Ethics statement

Written informed consents were obtained from the patients and their parents for publishing the pictures included in this manuscript.

## Author contributions

SD and ES drafted the initial manuscript, and revised and finalized the manuscript. FD and SO coordinated and supervised manuscript preparation, critically reviewed, and revised the manuscript. All authors approved the final manuscript as submitted.

### Conflict of interest statement

The authors declare that the research was conducted in the absence of any commercial or financial relationships that could be construed as a potential conflict of interest.

## References

[1] Ehrlich P Studies in Immunity. London: Wiley (1910).

[2] Masters SL Simon A Aksentijevich I Kastner DL. Horror autoinflammaticus: the molecular pathophysiology of autoinflammatory disease (^*^). Annu Rev Immunol. (2009) 27:621–68. 10.1146/annurev.immunol.25.022106.141627 19302049 PMC2996236

[3] Jennette JC Falk RJ Bacon PA Basu N Cid MC Ferrario F. 2012 revised International Chapel Hill Consensus Conference nomenclature of vasculitides. Arthritis Rheum. (2013) 65:1–11. 10.1002/art.37715 23045170

[4] Peleg H Ben-Chetrit E. Vasculitis in the autoinflammatory diseases. Curr Opin Rheumatol. (2017) 29:4–11. 10.1097/BOR.0000000000000347 27755121

[5] Ginsberg S Rosner I Rozenbaum M Slobodin G Zilber K Boulman N. Autoinflammatory associated vasculitis. Semin Arthritis Rheum. (2016) 46:367–71. 10.1016/j.semarthrit.2016.07.007 27612399

[6] Chen Y Li X Boini KM Pitzer AL Gulbins E Zhang Y. Endothelial Nlrp3 inflammasome activation associated with lysosomal destabilization during coronary arteritis. Biochim Biophys Acta (2015) 1853:396–408. 10.1016/j.bbamcr.2014.11.012 25450976 PMC4289419

[7] Xia M Boini KM Abais JM Xu M Zhang Y Li PL. Endothelial NLRP3 inflammasome activation and enhanced neointima formation in mice by adipokine 0visfatin. Am J Pathol. (2014) 184:1617–28. 10.1016/j.ajpath.2014.01.032 24631027 PMC4005976

[8] Kalyoncu M Acar BC Cakar N Bakkaloglu A Ozturk S Dereli E. Are carriers for MEFV mutations “healthy”? Clin Exp Rheumatol. (2006) 24(5 Suppl. 42):S120–2. 17067442

[9] Yalçinkaya F Ozen S Ozçakar ZB Aktay N Cakar N Düzova A. A new set of criteria for the diagnosis of familial Mediterranean fever in childhood. Rheumatology (2009) 48:395–8. 10.1093/rheumatology/ken509 19193696

[10] Barzilai A Langevitz P Goldberg I Kopolovic J Livneh A Pras M. Erysipelas-like erythema of familial Mediterranean fever: clinicopathologic correlation. J Am Acad Dermatol. (2000) 42(5 Pt 1):791–5. 10.1067/mjd.2000.103048 10775856

[11] Özçakar ZB Çakar N Uncu N Çelikel BA Yalçinkaya F. Familial Mediterranean fever-associated diseases in children. QJM (2017) 110:287–90. 10.1093/qjmed/hcw230 28040706

[12] Ozdogan H Arisoy N Kasapçapur O Sever L Calişkan S Tuzuner N. Vasculitis in familial Mediterranean fever. J Rheumatol. (1997) 24:323–7. 9034991

[13] Tekin M Yalçinkaya F Tümer N Akar N Misirlioglu M Cakar N. Clinical, laboratory and molecular characteristics of children with Familial Mediterranean Fever-associated vasculitis. Acta Paediatr. (2000) 89:177–82. 10.1111/j.1651-2227.2000.tb01212.x 10709887

[14] Tunca M Akar S Onen F Ozdogan H Kasapcopur O Yalcinkaya F. Familial Mediterranean fever (FMF) in Turkey: results of a nationwide multicenter study. Medicine (2005) 84:1–11. 10.1097/01.md.0000152370.84628.0c 15643295

[15] Bayram C Demircin G Erdogan O Bülbül M Caltik A Akyüz SG. Prevalence of MEFV gene mutations and their clinical correlations in Turkish children with Henoch-Schonlein purpura. Acta Paediatr. (2011) 100:745–9. 10.1111/j.1651-2227.2011.02143.x 21231959

[16] Dogan CS Akman S Koyun M Bilgen T Comak E Gokceoglu AU. Prevalence and significance of the MEFV gene mutations in childhood Henoch-Schonlein purpura without FMF symptoms. Rheumatol Int. (2013) 33:377–80. 10.1007/s00296-012-2400-x 22451026

[17] Ben-Chetrit E Yazici H. Non-thrombocytopenic purpura in familial Mediterranean fever-comorbidity with Henoch-Schonlein purpura or an additional rare manifestation of familial Mediterranean fever? Rheumatology (2016) 55:1153–8. 10.1093/rheumatology/kev378 26464521

[18] Ozen S Ben-Chetrit E Bakkaloglu A Gur H Tinaztepe K Calguneri M Polyarteritis nodosa in patients with Familial Mediterranean Fever (FMF): a concomitant disease or a feature of FMF? Semin Arthritis Rheum. (2001) 30:281–7. 10.1053/sarh.2001.19958 11182028

[19] Ozkaya O Bek K Alaca N Ceyhan M Açikgöz Y Taşdemir HA. Cerebral vasculitis in a child with Henoch-Schonlein purpura and familial Mediterranean fever. Clin Rheumatol. (2007) 26:1729–32. 10.1007/s10067-006-0485-x 17235658

[20] Luger S Harter PN Mittelbronn M Wagner M Foerch C. Brain stem infarction associated with familial Mediterranean fever and central nervous system vasculitis. Clin Exp Rheumatol. (2013) 31(3 Suppl. 77):93–5. 23710607

[21] Serrano R Martínez MA Andrés A Morales JM Samartin R. Familial mediterranean fever and acute myocardial infarction secondary to coronary vasculitis. Histopathology (1998) 33:163–7. 9762550 10.1046/j.1365-2559.1998.00462.x

[22] Zihni FY Kalfa M Ocakçi PT Tarhan F Parildar M Keser G. Coexistence of Takayasu's arteritis with familial Mediterranean fever. Rheumatol Int. (2012) 32:1675–8. 10.1007/s00296-011-1853-7 21416236

[23] Schlesinger M Kopolovic J Viskoper RJ Ron N. A case of familial Mediterranean fever with cutaneous vasculitis and immune complex nephritis: light, electron, and immunofluorescent study of renal biopsy. Am J Clin Pathol. (1983) 80:511–4. 10.1093/ajcp/80.4.511 6353905

[24] Jain A Misra DP Sharma A Wakhlu A Agarwal V Negi VS. Vasculitis and vasculitis-like manifestations in monogenic autoinflammatory syndromes. Rheumatol Int. (2018) 38:13–24. 10.1007/s00296-017-3839-6 29032440

[25] Sag E Bilginer Y Ozen S. Autoinflammatory Diseases with periodic fevers. Curr Rheumatol Rep. (2017) 19:41. 10.1007/s11926-017-0670-8 28631068

[26] Kolivras A Theunis A Ferster A Lipsker D Sass U Dussart A. Cryopyrin-associated periodic syndrome: an autoinflammatory disease manifested as neutrophilic urticarial dermatosis with additional perieccrine involvement. J Cutan Pathol. (2011) 38:202–8. 10.1111/j.1600-0560.2010.01638.x 21062341

[27] Ebrahimi-Fakhari D Wahlster L Mackensen F Blank N. Clinical manifestations and longterm followup of a patient with CINCA/NOMID syndrome. J Rheumatol. (2010) 37:2196–7. 10.3899/jrheum.100290 20889617

[28] Russo RA Katsicas MM. Chronic infantile neurological cutaneous and articular syndrome: two new cases with rare manifestations. Acta Paediatr. (2001) 90:1076–9. 10.1111/j.1651-2227.2001.tb01367.x 11683199

[29] Khemani C Khubchandani R. CINCA Syndrome. Indian Pediatr. (2007) 44:933–6. 18175851

[30] Ter Haar NM Jeyaratnam J Lachmann HJ Simon A Brogan PA Doglio M. The phenotype and genotype of mevalonate kinase deficiency: A series of 114 cases from the Eurofever Registry. Arthritis Rheumatol. (2016) 68:2795–805. 10.1002/art.39763 27213830

[31] Drenth JP Boom BW Toonstra J Van der Meer JW. Cutaneous manifestations and histologic findings in the hyperimmunoglobulinemia D syndrome. International Hyper IgD Study Group. Arch Dermatol. (1994) 130:59–65. 10.1001/archderm.1994.01690010063008 8285741

[32] van der Meer JW Vossen JM Radl J van Nieuwkoop JA Meyer CJ Lobatto S. Hyperimmunoglobulinaemia D and periodic fever: a new syndrome. Lancet (1984) 1:1087–10. 6144826 10.1016/s0140-6736(84)92505-4

[33] Boom BW Daha MR Vermeer BJ van der Meer JW. IgD immune complex vasculitis in a patient with hyperimmunoglobulinemia D and periodic fever. Arch Dermatol. (1990) 126:1621–4. 10.1001/archderm.1990.01670360085015 2147822

[34] Topaloglu R Saatci U. Hyperimmunoglobulinaemia D and periodic fever mimicking familial Mediterranean fever in the Mediterranean. Postgrad Med J. (1991) 67:490–1. 1852679 10.1136/pgmj.67.787.490-a PMC2398873

[35] Toro JR Aksentijevich I Hull K Dean J Kastner DL. Tumor necrosis factor receptor-associated periodic syndrome: a novel syndrome with cutaneous manifestations. Arch Dermatol. (2000) 136:1487–94. 10.1001/archderm.136.12.1487 11115159

[36] Lamprecht P Moosig F Adam-Klages S Mrowietz U Csernok E Kirrstetter M. Small vessel vasculitis and relapsing panniculitis in tumour necrosis factor receptor associated periodic syndrome (TRAPS) Ann Rheum Dis. (2004) 63:1518–20. 10.1136/ard.2003.016733 15479908 PMC1754814

[37] Hernández-Rodríguez J Ruíz-Ortiz E Tomé A Espinosa G González-Roca E Mensa-Vilaró A. Clinical and genetic characterization of the autoinflammatory diseases diagnosed in an adult reference center. Autoimmun Rev. (2016) 15:9–15. 10.1016/j.autrev.2015.08.008 26299986

[38] Cammelli D1 Vitiello G Troilo A Comin CE Cantarini L. Systemic vasculopathy in a patient with tumor necrosis factor receptor-associated periodic syndrome. J Clin Rheumatol. (2017) 23:395–7. 10.1097/RHU.0000000000000533 28937477

[39] Lindor NM Arsenault TM Solomon H Seidman CE McEvoy MT. A new autosomal dominant disorder of pyogenic sterile arthritis, pyoderma gangrenosum, and acne: PAPA syndrome. Mayo Clin Proc. (1997) 72:611–5. 10.1016/S0025-6196(11)63565-9 9212761

[40] Niv D Ramirez JA Fivenson DP. Pyoderma gangrenosum, acne, and hidradenitis suppurativa (PASH) syndrome with recurrent vasculitis. JAAD Case Rep. (2017) 3:70–3. 10.1016/j.jdcr.2016.11.006 28203623 PMC5294749

[41] Khatibi K Heit JJ Telischak NA Elbers JM Do HM3. Cerebral vascular findings in PAPA syndrome: cerebral arterial vasculopathy or vasculitis and a posterior cerebral artery dissecting aneurysm. J Neurointerv Surg. (2016) 8:e29 10.1136/neurintsurg-2015-011753 26122324

[42] Steiner A Harapas CR Masters SL Davidson S. An update on autoinflammatory diseases: relopathies. Curr Rheumatol Rep. (2018) 20:39. 10.1007/s11926-018-0749-x 29846841

[43] Aksentijevich I Zhou Q. NF-kappaB pathway in Autoinflammatory Diseases: dysregulation of protein modifications by ubiquitin defines a new category of Autoinflammatory Diseases. Front Immunol. (2017) 8:399 10.3389/fimmu.2017.00399 28469620 PMC5395695

[44] Peltzer N Rieser E Taraborrelli L Draber P Darding M Pernaute B. HOIP deficiency causes embryonic lethality by aberrant TNFR1-mediated endothelial cell death. Cell Rep. (2014) 9:153–165. 10.1016/j.celrep.2014.08.066 25284787

[45] Zhou Q Yu X Demirkaya E Deuitch N Stone D Tsai WL. Biallelic hypomorphic mutations in a linear deubiquitinase define otulipenia, an early-onset autoinflammatory disease. Proc Natl Acad Sci USA. (2016) 113:10127–32. 10.1073/pnas.1612594113 27559085 PMC5018768

[46] Zhou Q Wang H Schwartz DM Stoffels M Park YH Zhang Y. Loss-of-function mutations in TNFAIP3 leading to A20 haploinsufficiency cause an early-onset autoinflammatory disease. Nat Genet. (2016) 48:67–73. 10.1038/ng.3459 26642243 PMC4777523

[47] Aeschlimann FA Batu ED Canna SW Go E Gül A Hoffmann P. A20 haploinsufficiency (HA20): clinical phenotypes and disease course of patients with a newly recognised NF-kB-mediated autoinflammatory disease. Ann Rheum Dis. (2018) 77:728–35. 10.1136/annrheumdis-2017-212403 29317407

[48] Crow YJ Chase DS Lowenstein Schmidt J Szynkiewicz M Forte GM Gornall HL. Characterization of human disease phenotypes associated with mutations in TREX1, RNASEH2A, RNASEH2B, RNASEH2C, SAMHD1, ADAR, and IFIH1. Am J Med Genet A (2015) 167A:296–312. 10.1002/ajmg.a.36887 25604658 PMC4382202

[49] Liu Y Jesus AA Marrero B Yang D Ramsey SE Sanchez GAM. Activated STING in a vascular and pulmonary syndrome. N Engl J Med. (2014) 371:507–18. 10.1056/NEJMoa1312625 25029335 PMC4174543

[50] Torrelo A Patel S Colmenero I Gurbindo D Lendínez F Hernández A. Chronic atypical neutrophilic dermatosis with lipodystrophy and elevated temperature (CANDLE) syndrome. J Am Acad Dermatol. (2010) 62:489–95. 10.1016/j.jaad.2009.04.046 20159315

[51] Liu Y Ramot Y Torrelo A Paller AS Si N Babay S. Mutations in proteasome subunit β type 8 cause chronic atypical neutrophilic dermatosis with lipodystrophy and elevated temperature with evidence of genetic and phenotypic heterogeneity. Arthritis Rheum. (2012) 64:895–907. 10.1002/art.33368 21953331 PMC3278554

[52] Torrelo A Colmenero I Requena L Paller AS Ramot Y Richard Lee CC. Histologic and Immunohistochemical Features of the Skin Lesions in CANDLE Syndrome. Am J Dermatopathol. (2015) 37:517–22. 10.1097/DAD.0000000000000340 26091509 PMC4476069

[53] Volpi S Tsui J Mariani M Pastorino C Caorsi R Sacco O. Type I interferon pathway activation in COPA syndrome. Clin Immunol. (2018) 187:33–6. 10.1016/j.clim.2017.10.001 29030294

[54] Watkin LB Jessen B Wiszniewski W Vece TJ Jan M Sha Y. COPA mutations impair ER-Golgi transport and cause hereditary autoimmune-mediated lung disease and arthritis. Nat Genet. (2015) 47:654–60. 10.1038/ng.3279 25894502 PMC4513663

[55] Vece TJ Watkin LB Nicholas S Canter D Braun MC Guillerman RP. Copa syndrome: a novel autosomal dominant immune Dysregulatory Disease. J Clin Immunol. (2016) 36:377–87. 10.1007/s10875-016-0271-8 27048656 PMC4842120

[56] Sanchez GAM Reinhardt A Ramsey S Wittkowski H Hashkes PJ Berkun Y. JAK1/2 inhibition with baricitinib in the treatment of autoinflammatory interferonopathies. J Clin Invest. (2018) 128:3041–52. 10.1172/JCI98814 29649002 PMC6026004

[57] Aksentijevich I Masters SL Ferguson PJ Dancey P Frenkel J van Royen-Kerkhoff A. An autoinflammatory disease with deficiency of the interleukin-1-receptor antagonist. N Engl J Med. (2009) 360:2426–37. 10.1056/NEJMoa0807865 19494218 PMC2876877

[58] Matsuki T Isoda K Horai R Nakajima A Aizawa Y Suzuki K. Involvement of tumor necrosis factor-alpha in the development of T cell-dependent aortitis in interleukin-1 receptor antagonist-deficient mice. Circulation (2005) 112:1323–31. 10.1161/CIRCULATIONAHA.105.564658 16129814

[59] Vettiyil G Punnen A Kumar S. An unusual association of chronic recurrent multifocal osteomyelitis, pyoderma gangrenosum, and takayasu arteritis. J Rheumatol. (2017) 44:127–8. 10.3899/jrheum.160491 28042129

[60] Pelkonen P Ryöppy S Jääskeläinen J Rapola J Repo H Kaitila I. Chronic osteomyelitislike disease with negative bacterial cultures. Am J Dis Child. (1988) 142:1167–73. 3177323 10.1001/archpedi.1988.02150110045017

[61] Behcet H Über rezidivierende, aphtöse, durch ein virus verursachte geschwüre am mund, am auge und an den genitalien. Dermatol Wochensch. (1937) 105:1152–63.

[62] Dilsen N. History and development of Behcet's disease. Rev Rhum Engl Ed. (1996) 63:512–9. 8896069

[63] Yurdakul S Yazici Y Epidemiology of Behçet's syndrome and regional differences in disease expression. In: Yazici YH, editor. Behcet's Syndrome. New York, NY: Springer (2010). p. 35–53.

[64] Kural-Seyahi E Fresko I Seyahi N Ozyazgan Y Mat C Hamuryudan V. The long-term mortality and morbidity of Behcet syndrome: a 2-decade outcome survey of 387 patients followed at a dedicated center. Medicine (2003) 82:60–76. 10.1097/00005792-200301000-00006 12544711

[65] Criteria for diagnosis of Behcet's disease International Study Group for Behcet's Disease. Lancet (1990) 335:1078–80. 1970380

[66] Koné-Paut I Shahram F Darce-Bello M Cantarini L Cimaz R Gattorno M. Consensus classification criteria for paediatric Behcet's disease from a prospective observational cohort: PEDBD. Ann Rheum Dis. (2016) 75:958–64. 10.1136/annrheumdis-2015-208491 26698843

[67] Batu ED Sönmez HE Sözeri B Butbul Aviel Y Bilginer Y Özen S. The performance of different classification criteria in paediatric Behcet's disease. Clin Exp Rheumatol. (2017) 35(Suppl. 108):119–23. 28406761

[68] Hatemi G Meara A Ozguler Y Direskeneli H Mahr A Easley E. Developing a core set of outcome measures for behcet disease: report from OMERACT (2016). J Rheumatol. (2017) 44:1750–53. 10.3899/jrheum.161352 28365574 PMC7217335

[69] Ozen S Frenkel J Ruperto N Gattorno M. The Eurofever Project: towards better care for autoinflammatory diseases. Eur J Pediatr. (2011) 170:445–52. 10.1007/s00431-011-1411-z 21360011

[70] Gul A. Pathogenesis of Behcet's disease: autoinflammatory features and beyond. Semin Immunopathol. (2015) 37:413–8. 10.1007/s00281-015-0502-8 26068404

[71] Ambrose N Khan E Ravindran R Lightstone L Abraham S Botto M. The exaggerated inflammatory response in Behcet's syndrome: identification of dysfunctional post-transcriptional regulation of the IFN-gamma/CXCL10 IP-10 pathway. Clin Exp Immunol. (2015) 181:427–33. 10.1111/cei.12655 25982097 PMC4557378

[72] de Menthon M Lavalley MP Maldini C Guillevin L Mahr A. HLA-B51/B5 and the risk of Behcet's disease: a systematic review and meta-analysis of case-control genetic association studies. Arthritis Rheum. (2009) 61:1287–96. 10.1002/art.24642 19790126 PMC3867978

[73] Mizuki N Meguro A Ota M Ohno S Shiota T Kawagoe T. Genome-wide association studies identify IL23R-IL12RB2 and IL10 as Behcet's disease susceptibility loci. Nat Genet. (2010) 42:703–6. 10.1038/ng.624 20622879

[74] Fei Y Webb R Cobb BL Direskeneli H Saruhan-Direskeneli G Sawalha AH. Identification of novel genetic susceptibility loci for Behcet's disease using a genome-wide association study. Arthritis Res Ther. (2009) 11:R66. 10.1186/ar2695 19442274 PMC2714112

[75] Remmers EF Cosan F Kirino Y Ombrello MJ Abaci N Satorius C. Genome-wide association study identifies variants in the MHC class I, IL10, and IL23R-IL12RB2 regions associated with Behcet's disease. Nat Genet. (2010) 42:698–702. 10.1038/ng.625 20622878 PMC2923807

[76] Kirino Y Bertsias G Ishigatsubo Y Mizuki N Tugal-Tutkun I Seyahi E. Genome-wide association analysis identifies new susceptibility loci for Behcet's disease and epistasis between HLA-B^*^51 and ERAP1. Nat Genet. (2013) 45:202–7. 10.1038/ng.2520 23291587 PMC3810947

[77] Lee YJ Horie Y Wallace GR Choi YS Park JA Choi JY. Genome-wide association study identifies GIMAP as a novel susceptibility locus for Behcet's disease. Ann Rheum Dis. (2013) 72:1510–6. 10.1136/annrheumdis-2011-200288 23041938

[78] Hou S Yang Z Du L Jiang Z Shu Q Chen Y. Identification of a susceptibility locus in STAT4 for Behcet's disease in Han Chinese in a genome-wide association study. Arthritis Rheum. (2012) 64:4104–13. 10.1002/art.37708 23001997

[79] DeLay ML Turner MJ Klenk EI Smith JA Sowders DP Colbert RA. HLA-B27 misfolding and the unfolded protein response augment interleukin-23 production and are associated with Th17 activation in transgenic rats. Arthritis Rheum. (2009) 60:2633–43. 10.1002/art.24763 19714651 PMC2893331

[80] Ozen S Eroglu FK. Pediatric-onset Behcet disease. Curr Opin Rheumatol. (2013) 25:636–42. 10.1097/BOR.0b013e328363ea8b 23872902

[81] Ozen S Acar-Ozen NP. Recent advances in childhood vasculitis. Curr Opin Rheumatol. (2017) 29:530–4. 10.1097/BOR.0000000000000424 28582318

[82] Tascilar K Melikoglu M Ugurlu S Sut N Caglar E Yazici H. Vascular involvement in Behcet's syndrome: a retrospective analysis of associations and the time course. Rheumatology (2014) 53:2018–22. 10.1093/rheumatology/keu233 24907156

[83] Seyahi E Melikoglu M Akman C Hamuryudan V Ozer H Hatemi G. Pulmonary artery involvement and associated lung disease in Behcet disease: a series of 47 patients. Medicine (2012) 91:35–48. 10.1097/MD.0b013e318242ff37 22210555

[84] Seyahi E Caglar E Ugurlu S Kantarci F Hamuryudan V Sonsuz A. An outcome survey of 43 patients with Budd-Chiari syndrome due to Behcet's syndrome followed up at a single, dedicated center. Semin Arthritis Rheum. (2015) 44:602–9. 10.1016/j.semarthrit.2014.10.014 25476470

[85] Khan E Ambrose NL Ahnström J Kiprianos AP Stanford MR Eleftheriou D. A low balance between microparticles expressing tissue factor pathway inhibitor and tissue factor is associated with thrombosis in Behcet's Syndrome. Sci Rep. (2016) 6:38104. 10.1038/srep38104 27924945 PMC5141484

[86] Tohme A Aoun N El-Rassi B Ghayad E. Vascular manifestations of Behçet's disease. Eighteen cases among 140 patients. Joint Bone Spine (2003) 70:384–9. 10.1016/S1297-319X(03)00076-9 14563470

[87] Sakane T Takeno M Suzuki N Inaba G. Behcet's disease. N Engl J Med. (1999) 341:1284–91. 10.1056/NEJM199910213411707 10528040

[88] Seyahi E Cakmak OS Tutar B Arslan C Dikici AS Sut N. Clinical and ultrasonographic evaluation of lower-extremity vein thrombosis in behcet syndrome: an observational study. Medicine (Baltimore) (2015) 94:e1899. 10.1097/MD.0000000000001899 26554787 PMC4915888

[89] Uluduz D Kürtüncü M Yapici Z Seyahi E Kasapçopur Ö Özdogan H. Clinical characteristics of pediatric-onset neuro-Behcet disease. Neurology (2011) 77:1900–5. 10.1212/WNL.0b013e318238edeb 22076549

[90] Krause I Leibovici L Guedj D Molad Y Uziel Y Weinberger A. Disease patterns of patients with Behcet's disease demonstrated by factor analysis. Clin Exp Rheumatol. (1999) 17:347–50. 10410270

[91] Seyahi E Yurdakul S. Behcet's Syndrome and Thrombosis. Mediterr J Hematol Infect Dis. (2011) 3:e2011026. 10.4084/MJHID.2011.026 21869912 PMC3152448

[92] Seyahi E. Behcet's disease: how to diagnose and treat vascular involvement. Best Pract Res Clin Rheumatol. (2016) 30:279–95. 10.1016/j.berh.2016.08.002 27886800

[93] Saadoun D Asli B Wechsler B Houman H Geri G Desseaux K. Long-term outcome of arterial lesions in Behcet disease: a series of 101 patients. Medicine (Baltimore) (2012) 91:18–24. 10.1097/MD.0b013e3182428126 22198498

[94] Mogulkoc N Burgess MI Bishop PW. Intracardiac thrombus in Behcet's disease: a systematic review. Chest (2000) 118:479–87. 10.1378/chest.118.2.479 10936144

[95] Yazici H Ugurlu S Seyahi E. Behcet syndrome: is it one condition? Clin Rev Allergy Immunol. (2012) 43:275–80. 10.1007/s12016-012-8319-x 22674015

[96] Tunc R Keyman E Melikoglu M Fresko I Yazici H. Target organ associations in Turkish patients with Behcet's disease: a cross sectional study by exploratory factor analysis. J Rheumatol. (2002) 29:2393–6. 12415598

[97] Hatemi G Christensen R Bang D Bodaghi B Celik AF Fortune F. 2018 update of the EULAR recommendations for the management of Behcet's syndrome. Ann Rheum Dis. (2018) 77:808–18. 10.1136/annrheumdis-2018-213225 29625968

